# Successful Treatment of Osimertinib Resistance in an EGFR‐Mutant Lung Cancer Patient With a Rare STRN3‐ALK Fusion Using Brigatinib and Osimertinib

**DOI:** 10.1002/kjm2.70021

**Published:** 2025-05-08

**Authors:** Chia‐Yu Kuo, Wei‐An Lai, Tai‐Huang Lee, Chih‐Jen Yang

**Affiliations:** ^1^ Division of Pulmonary and Critical Care Medicine, Department of Internal Medicine Kaohsiung Medical University Hospital, Kaohsiung Medical University Kaohsiung Taiwan; ^2^ Department of Pathology Kaohsiung Medical University Kaohsiung Taiwan

To the Editor,

Non‐small cell lung cancer (NSCLC) remains a leading cause of cancer mortality worldwide. Epidermal growth factor receptor (EGFR) mutations, particularly exon 19 deletion and L858R mutations, have transformed the treatment landscape of NSCLC, making tyrosine kinase inhibitors (TKIs) the standard of care [1]. Osimertinib, a third‐generation EGFR TKI, has demonstrated efficacy in patients with EGFR T790M mutation after progression on first‐ or second‐generation EGFR TKIs [2]. However, acquired resistance to osimertinib is inevitable, with mechanisms such as EGFR‐dependent mutations, MET amplification, and ALK rearrangements being implicated [3]. Here, we report a rare case of osimertinib resistance mediated by STRN3‐ALK fusion and successfully managed with a combination of osimertinib and brigatinib.

A 70‐year‐old never‐smoking female with EGFR‐mutant (exon 19 deletion) lung adenocarcinoma was diagnosed at stage IVA (cT4N2M1a). She was initially treated with afatinib (30 mg daily) and demonstrated tumor shrinkage. However, disease progression with pleural effusion occurred after 13 months. Cytological analysis of pleural fluid confirmed adenocarcinoma, and real‐time PCR identified the EGFR exon 19 deletion with acquired T790M mutation. Consequently, osimertinib (80 mg daily) was initiated. After 3 months, disease progression was observed with increased tumor burden and worsening pleural effusion (Figure 1A). Next‐generation sequencing (NGS) of pleural fluid identified EGFR exon 19 deletion, TP53 mutation, and an STRN3‐ALK fusion. Immunohistochemistry confirmed ALK rearrangement (Figure 1C,D). Given the emergence of the STRN3‐ALK fusion as a resistance mechanism to osimertinib, a combination therapy of osimertinib (80 mg daily) and brigatinib (180 mg daily) was administered. The patient tolerated the combination well without significant toxicities. After 3 months, chest CT demonstrated partial tumor response per RECIST 1.1 (Figure 1B).

**FIGURE 1:**
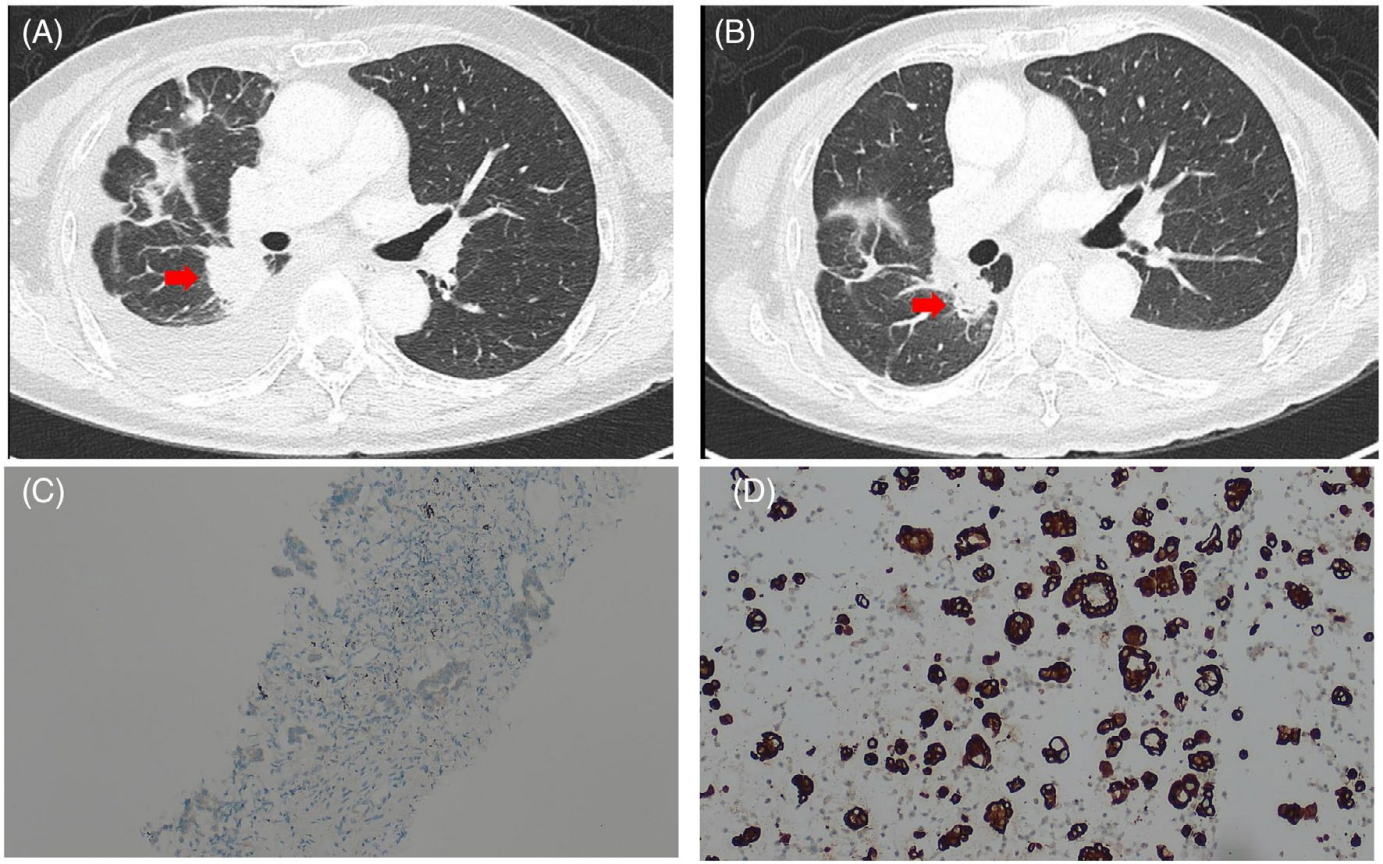
(A) Lung cancer progression observed under treatment with osimertinib (80 mg). (B) Partial response observed after 3 months of combination therapy with osimertinib and brigatinib. (C) Negative for ALK by immunohistochemical stain of clone D5F3 in the initial tissue specimen (100× magnification). (D) Positive for ALK by immunohistochemical stain of clone D5F3 in pleural effusion (100× magnification).

The STRN3‐ALK fusion is an extremely rare genetic alteration that has been reported in only a few cases of NSCLC. Unlike the more common EML4‐ALK fusion, the STRN3‐ALK fusion has unique oncogenic properties and remains poorly understood. The STRN family of proteins, including STRN3, has been implicated in intracellular signaling pathways that promote tumor growth and metastasis [4]. The fusion of STRN3 with ALK may lead to constitutive activation of ALK kinase activity, driving resistance to EGFR TKIs and promoting tumor progression. Given the rarity of the STRN3‐ALK fusion, optimal treatment strategies remain uncertain, necessitating further research into its functional significance and targeted therapies.

Acquired ALK rearrangement as a resistance mechanism to osimertinib is exceedingly rare (< 1%). Previous reports have described EML4‐ALK fusions emerging post‐osimertinib resistance, with combination TKI therapy yielding favorable outcomes. EML4‐ALK fusions are rarer but have been implicated in NSCLC progression and metastasis [5]. Brigatinib, a second‐generation ALK TKI, has been shown to be effective against ALK‐rearranged NSCLC and may provide a rational treatment option when ALK rearrangement emerges as a resistance mechanism to osimertinib. The combination strategy targeting both EGFR and ALK pathways aims to suppress tumor proliferation and delay further resistance mechanisms.

The identification of STRN3‐ALK fusion highlights the need for routine molecular profiling in NSCLC patients who develop resistance to EGFR TKIs. While osimertinib remains a cornerstone in EGFR‐mutant NSCLC, its long‐term efficacy is often hindered by resistance mechanisms. The use of next‐generation sequencing allows for precise identification of novel resistance alterations, ensuring that patients receive appropriate targeted therapies. The success of brigatinib and osimertinib combination therapy in this case suggests that dual inhibition strategies may be viable options for overcoming resistance mediated by STRN3‐ALK fusion. Further clinical trials are warranted to determine the optimal therapeutic approach for patients with this rare fusion.

This case underscores the importance of repeat molecular testing upon disease progression to identify actionable resistance mechanisms. Early identification of ALK fusions through NGS allows for prompt intervention with appropriate targeted therapy. Future studies should explore the role of combination targeted therapy in managing ALK‐mediated resistance in EGFR‐mutant NSCLC, potentially leading to novel therapeutic paradigms.

## Conflicts of Interest

The authors declare no conflicts of interest.

## Data Availability

The data that support the findings of this study are available on request from the corresponding author. The data are not publicly available due to privacy or ethical restrictions.
